# Correction: Xia et al. The Kelch Repeat Protein VdKeR1 Is Essential for Development, Ergosterol Metabolism, and Virulence in *Verticillium dahliae*. *J. Fungi* 2024, *10*, 643

**DOI:** 10.3390/jof12070508

**Published:** 2026-07-10

**Authors:** Wen-Li Xia, Zhe Zheng, Feng-Mao Chen

**Affiliations:** Co-Innovation Center for Sustainable Forestry in Southern China, College of Forestry and Grassland, College of Soil and Water Conservation, Nanjing Forestry University, Nanjing 210037, China; xiaxia@njfu.edu.cn (W.-L.X.); zhengzhe@njfu.edu.cn (Z.Z.)

In the original publication [1], there were mistakes in Figures 3, 4 and 7. In each figure, there was a duplicated panel due to an error during figure assembly. The authors contacted the Editorial Office to request a Correction. The corrected Figure 3, Figure 4 and Figure 7 appear below.

The authors state that the scientific conclusions are unaffected. This correction was approved by the Academic Editor. The original publication has also been updated.

## Figures and Tables

**Figure 3:**
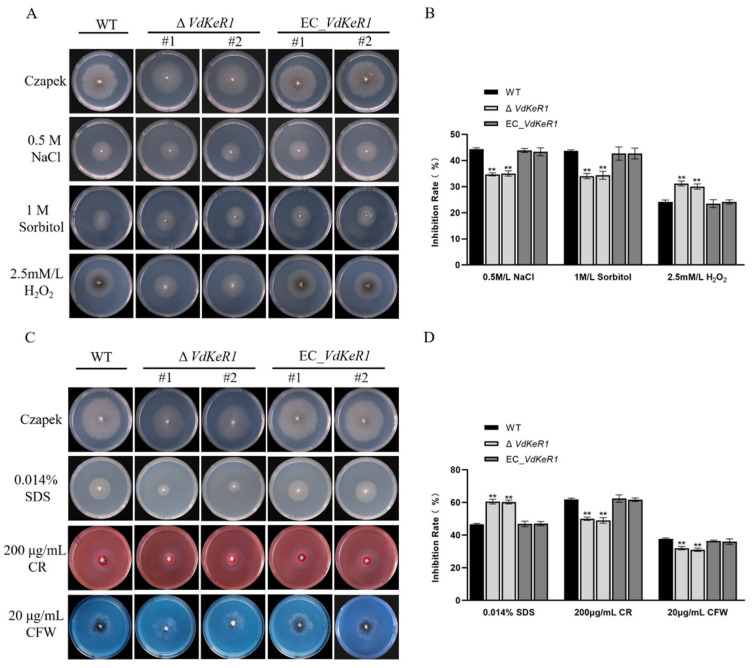
Effect of *VdKeR1* gene on sensitivity to osmotic stress, oxidative stress, and cell wall stress in *V. dahliae*. (**A**) Growth of each strain under different osmotic and oxidative stress factors. (**B**) The inhibition rate of each strain under different osmotic and oxidative stress factors. (**C**) The growth of each strain under different cell wall stress chemicals. (**D**) The inhibition rate of each strain under different cell wall stress chemicals. ** Δ*VdKeR1* was significantly different from WT and complemented strains (*p* < 0.01).

**Figure 4:**
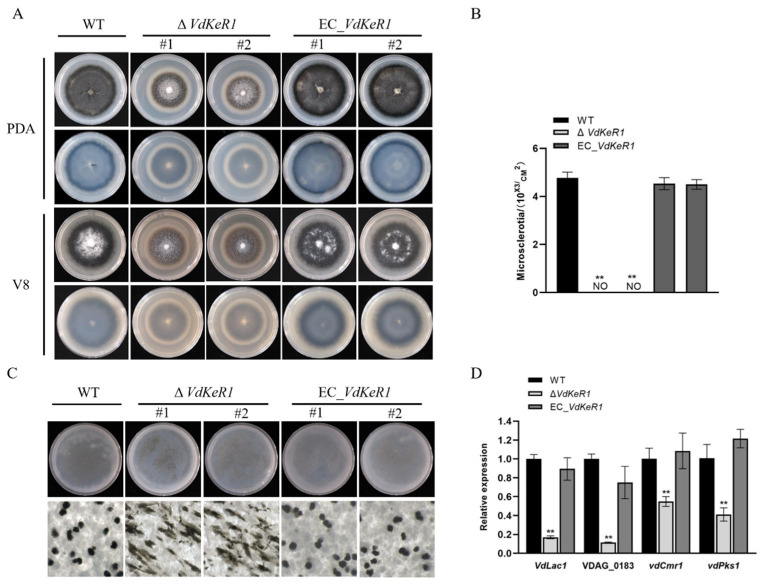
Effect of *VdKeR1* gene on melanin synthesis and microsclerotia formation in *V. dahliae*. (**A**) Melanin synthesis on V8 and PDA media. (**B**) Formation of microsclerotia. The spore suspension of each strain was spread on BMM medium covered with glass paper. (scale bar = 50 µm) (**C**) Micronucleus count statistics. The photographs in the bottom row of (**C**) were entered into Image J 1.5 software to obtain the number of particles per unit area. (**D**) Expression levels of genes related to microsclerotia formation and melanin synthesis. Gene expression analysis method as described in M and M. ** Δ*VdKeR1* was significantly different from WT and complemented strains (*p* < 0.01).

**Figure 7:**
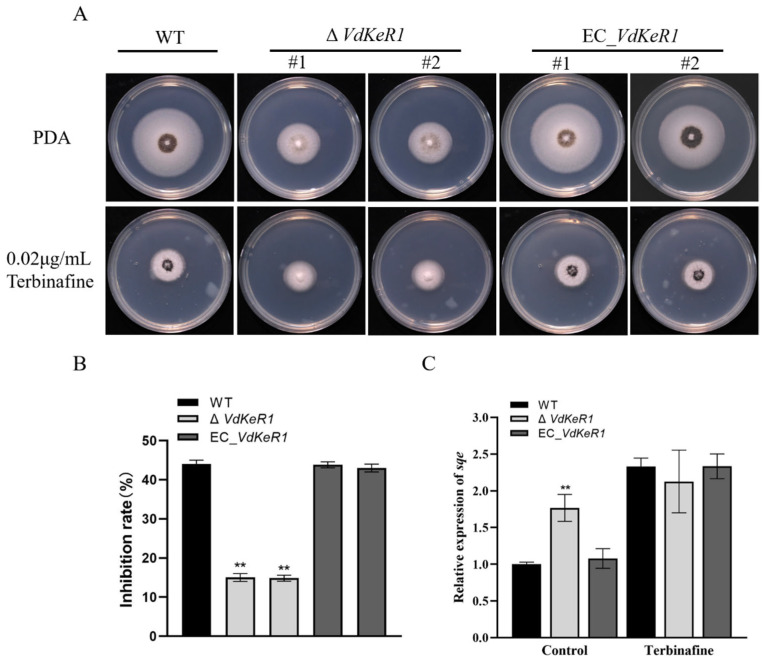
Response of WT, deletion mutant, and complemented strains l to terbinafine. (**A**) Growth of *V. dahliae* strains on PDA plates with and without terbinafine. (**B**) The inhibition rate of each strain under 0.02 μg/mL terbinafine. (**C**) qPCR was used to detect the expression levels of *sqe* gene in the absence and presence of terbinafine. Gene expression analysis method as described in M and M. ** Δ*VdKeR1* was significantly different from WT and complemented strains (*p* < 0.01).
