# Supplementary material for: The Kelch Repeat Protein VdKeR1 Is Essential for Development, Ergosterol Metabolism, and Virulence in Verticillium dahliae
Source: J Fungi (Basel). 2024 Sep 9;10(9):643. doi: 10.3390/jof10090643 (PMC11433423; doi:10.3390/jof10090643)
Supplement: Supplementary file 1 [file jof-10-00643-s001.zip › Figure S1. Construction and Validation of VdKeR1 Knockout Mutants and Complementary Strains .pdf]

3.2. Construction and Validation of VdKeR1 Knockout Mutants and Complementary Strains

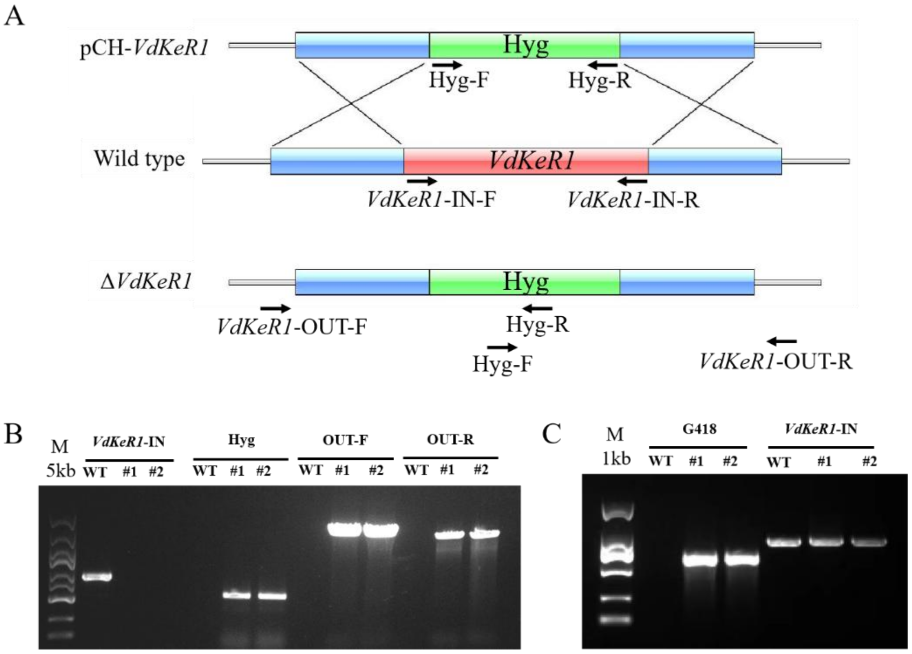

Figure S1. (A) Schematic diagram of the construction of a knockout vector using homologous recombination principle to replace *VdKeR1* gene with the hygromycin resistance gene (Hyg). (B) PCR identification of knockout transformants. The PCR identification was performed using *VdKeR1*-IN-F and *VdKeR1*-IN-R primers, Hyg-F and Hyg-R primers, *VdKeR1*-OUT-F and Hyg-R primers, Hyg-F and *VdKeR1*-OUT-R primers designed internally for the *VdKeR1* gene. (C) PCR identification of the complementary transformants; PCR identification was performed using G418-F and G418-R primers, as well as *VdKeR1*-IN-F and *VdKeR1*-IN-R primers.
