# Supplementary material for: The Kelch Repeat Protein VdKeR1 Is Essential for Development, Ergosterol Metabolism, and Virulence in Verticillium dahliae
Source: J Fungi (Basel). 2024 Sep 9;10(9):643. doi: 10.3390/jof10090643 (PMC11433423; doi:10.3390/jof10090643)
Supplement: Supplementary file 1 [file jof-10-00643-s001.zip › Tables S2-S7 mean values±SD.pdf]

Table S2. The effect of *VdKeR1* gene on the normal growth and the production of conidia in *V. dahliae.*

	WT	Δ*VdKeR1* #1	Δ*VdKeR1* #2	EC_*VdKeR1* #1	EC_*VdKeR1* #2
Growth diameter (mm) (PDA)	32.60±0.36	22.17±0.29**	22.43±0.51**	33.23±0.25	33.5±0.50
Conidiospore (10^5/cm^2)	3.15±0.35	0.20±0.02**	0.25±0.05**	3.24±0.21	3.22±0.19
Growth diameter (mm) (Czapek)	42.50±0.50	29.67±0.58**	29.83±0.76**	42.83±0.29	42.17±0.29
Growth diameter (mm) (Starch)	43.33±0.58	32.33±0.58**	32.83±0.76**	44.17±1.04	43.83±0.76
Growth diameter (mm) (Fructose)	30.67±1.15	25.50±0.50**	25.17±0.76**	31.00±1.00	30.23±0.40
Growth diameter (mm) (D-galactose)	12.83±0.29	12.83±0.29	13.00±0.50	12.50±0.50	12.50±0.50

The data is presented as the mean ± standard deviation (n=3). ** $P < 0.01$ in the table indicates a significant difference between △*VdKeR1* and WT as well as EC_*VdKeR1*.

Table S3. Effect of *VdKeR1* gene on sensitivity to osmotic stress, oxidative stress, and cell wall stress in *V. dahliae*.

Inhibition rate (%)	WT	Δ*VdKeR1* #1	Δ*VdKeR1* #2	EC_*VdKeR1* #1	EC_*VdKeR1* #2
0.5 M/L NaCl	44.33±0.58	34.67±0.58**	35.00±1.00**	43.83±0.76	43.33±1.53
1 M/L Sorbitol	43.70±0.40	34.00±1.00**	34.33±1.53**	42.67±2.52	42.67±2.08
2.5 mM/L H_2O_2	24.14±0.75	31.20±0.94**	30.00±1.00**	23.48±1.50	24.15±0.79
0.014% SDS	46.65±0.52	60.59±1.47**	60.26±1.19**	46.88±1.63	47.01±1.34
200 μg/mL CR	61.82±0.79	50.00±10.00**	49.00±1.73**	62.41±2.26	61.74±1.12
20 μg/mL CFW	37.67±0.58	32.00±1.00**	31.00±1.00**	36.33±0.58	36.00±1.73

The data is presented as the mean ± standard deviation (n=3). ** $P < 0.05$ in the table indicates a significant difference between △*VdKeR1* and WT as well as EC_*VdKeR1*.

The data is presented as the mean ± standard deviation (n=3). ** $P < 0.01$ in the table indicates

Table S4. Effect of *VdKeR1* gene on melanin synthesis and microsclerotia formation in *V. dahliae.*

	WT	Δ*VdKeR1*		EC_*VdKeR1*	
		#1	#2	#1	#2
Microsclerotia(10^3/cm^2)	4.77±0.25	0.00±0.00**	0.00±0.00**	4.53±0.25	4.5±0.20
Relative expression					
VdLac1	1.00±0.05	0.17±0.02**		0.89±0.12	
VDAG_0183	1.00±0.05	0.11±0.01**		0.75±0.17	
VdCmr1	1.00±0.11	0.55±0.05**		1.09±0.19	
VdPks1	1.01±0.15	0.41±0.07**		1.22±0.10	

a significant difference between △*VdKeR1* and WT as well as EC_*VdKeR1*.

Table S5. Effect of *VdKeR1* gene on the pathogenic process of *V. dahliae.*

	WT	Δ*VdKeR1* #1	Δ*VdKeR1* #2	EC_*VdKeR1* #1	EC_*VdKeR1* #2
Disease grade of cotton	81.00±0.03	4.00±0.01**	3.00±0.01**	81.00±0.03	78.00±0.03
Relative expression of biomass	1.01±0.13	0.02±0.01**	0.03±0.01**	1.16±0.03	1.06±0.05
Disease grade of *Acer truncatum*	77.00±0.04	6.00±0.01**	6.00±0.01**	75.00±0.05	76.00±0.03
Relative expression of biomass	1.01±0.16	0.05±0.01**	0.03±0.01**	1.15±0.25	1.18±0.11

The data is presented as the mean ± standard deviation (n=3). ** $P < 0.01$ in the table indicates a significant difference between △*VdKeR1* and WT as well as EC_*VdKeR1*.

The data is presented as the mean ± standard deviation (n=3). ** $P < 0.01$ in the table indicates

Table S6. Response of *V. dahliae* lacking *VdKeR1* gene to terbinafine.

	WT	Δ*VdKeR1*		EC_*VdKeR1*	
		#1	#2	#1	#2
Inhibition rate (%)	44.00±1.00	15.00±1.00**	14.83±0.76**	43.83±0.76	43.00±1.00
Relative expression of *sqe* (Control)	1.00±0.03	1.77±0.18**		1.08±0.13	
Relative expression of *sqe* (Terbinafine)	2.33±0.11	2.13±0.43		2.33±0.17	

a significant difference between △*VdKeR1* and WT as well as EC_*VdKeR1*.

The data is presented as the mean ± standard deviation (n=3). ** $P < 0.01$ in the table indicates a significant difference between △*VdKeR1* and WT as well as EC_*VdKeR1*.

Table S7. Effect of *VdKeR1* gene on squalene and ergosterol content in *V. dahliae.*

Content (mg/L)	WT	Δ*VdKeR1*	EC_*VdKeR1*
Squalene content (Control)	1.70±0.20	6.43±0.81**	1.86±0.25
Squalene content (Terbinafine)	17.00±1.00	7.50±0.30**	17.96±0.85
Ergosterol content (Control)	41.60±1.39	15.80±0.70**	42.50±0.50
Ergosterol content (Terbinafine)	0.81±0.09	10.51±0.44**	0.63±0.09
